# Managing Small Airway Disease in Patients with Severe Asthma: Transitioning from the “Silent Zone” to Achieving “Quiet Asthma”

**DOI:** 10.3390/jcm13082320

**Published:** 2024-04-17

**Authors:** Giovanna Elisiana Carpagnano, Andrea Portacci, Silvano Dragonieri, Francesca Montagnolo, Ilaria Iorillo, Ernesto Lulaj, Leonardo Maselli, Enrico Buonamico, Vitaliano Nicola Quaranta

**Affiliations:** Respiratory Diseases, University of Bari, 70121 Bari, Italy; elisiana.carpagnano@uniba.it (G.E.C.); a.portacci01@gmail.com (A.P.); francescamontagnolo17@gmail.com (F.M.); iorillo.ilaria@gmail.com (I.I.); ernestolulaj@gmail.com (E.L.); leonardomaselli@gmail.com (L.M.); enricobuonamico@gmail.com (E.B.); vitalianonicola.40@gmail.com (V.N.Q.)

**Keywords:** single-inhaler triple therapy, small airways disease, severe asthma

## Abstract

**Background/Objectives:** Several studies have demonstrated the positive clinical and functional impact of adding Long-Acting Muscarinic Antagonist (LAMA) to Inhaled Corticosteroids (ICS) and Long-Acting Beta-Agonists (LABA) therapy in the treatment of severe asthma. Aim and objectives: To demonstrate that treating Small Airways Disease (SAD) in severe asthma patients who are candidates for biologics can improve respiratory symptoms, lung function, and airways inflammation, potentially avoiding or delaying the use of biological therapy. **Methods:** Thirty-two severe asthma patients with SAD were transitioned from separate inhalers for ICS/LABA and LAMA to extrafine single-inhaler beclomethasone, formoterol, and glycopyrronium. None of these patients underwent biological therapy before the study. Follow-up evaluations were conducted at baseline (T0) and three months after initiation (T3). Assessments included clinical evaluations, spirometry, oscillometry, and inflammation markers. **Results:** Transitioning to single-inhaler triple therapy from T0 to T3 resulted in significant improvements in Asthma Control Test (ACT) and SAD parameters, including increased Forced Expiratory Volume in the mid-range of lung capacity and improved airway resistance and reactance measurements using impulse oscillometry. A significant reduction in airway inflammation was evidenced by lower levels of Fractional Exhaled Nitric Oxide 350 (FeNO 350) (*p* < 0.001 for all). **Conclusions**: Adopting a single-inhaler triple therapy notably enhanced clinical control and small airway function in patients with severe asthma and SAD, supporting the positive impact of target-therapy for the achievement of a stable state termed “Quiet Asthma”.

## 1. Introduction

Asthma is a highly prevalent chronic condition, affecting approximately 300 million individuals globally [1]. Severe asthma, constituting less than 10% of the total asthma population, disproportionately contributes to asthma care costs. This condition places a significant economic burden on healthcare systems, with associated costs experiencing a notable rise in recent years.

Effectively managing symptoms and minimizing the risk of future exacerbations are the key objectives in proper asthma care, aiming to preserve lung function [2].

A review and meta-analysis involving 20 randomized clinical trials with 11,894 patients showed that triple therapy inhaled corticosteroids (ICS), long-acting β2-agonists (LABA), and long-acting muscarinic antagonists (LAMA)) compared to dual therapy (ICS plus LABA) significantly reduced severe asthma exacerbations (risk ratio, 0.83) and slightly improved asthma control [3].

LAMAs offer a unique method of bronchodilation different from LABAs [4], making them a beneficial addition for persistent, uncontrolled asthma. Including LAMAs in a treatment regimen with ICS and LABAs may help achieve optimal asthma control. This approach could prevent the need for escalating to more aggressive treatments like oral corticosteroids, which have a higher risk of adverse effects [5], or biologics, which significantly increase treatment costs [6].

The TRIMARAN and TRIGGER trials, both phase 3, double-blind, randomized, active-controlled studies, focused on the efficacy of single inhaler extra-fine triple therapy in uncontrolled asthma [7]. These studies demonstrated that the addition of LAMA within a single inhalation device, combined with the existing treatment of ICS and LABA, significantly improved lung function and reduced exacerbation rates in patients suffering from uncontrolled asthma [7].

The effectiveness of asthma treatment is partly due to the synergistic effect of combining LAMA, LABA, and ICS in a single device. The suggestion that combining LAMAs with ICS or ICS-Long-LABA may result in synergistic effects is primarily based on research conducted using animal models and in vitro studies [8].

Another possible explanation is the ability to target the small airways as in extra-fine formulation. Previous studies indicate that persistent inflammation can cause small airway dysfunction (SAD), adversely affecting asthma control. According to Atlantis study, a significant proportion of the asthmatic cohort, accounting for 91%, presents with a phenotype characteristic of small airway disease. This underscores the clinical relevance of utilizing extra-fine formulations as a targeted therapeutic approach in precision medicine for this patient population [9].

Effective asthma management relies on controlling the small airways, with Impulse Oscillometry (IOS) proving to be an efficient and simple method for SAD. Outperforming traditional spirometry, IOS is particularly adept at evaluating small airway function and identifying SAD. Thanks to its heightened sensitivity, IOS serves as a crucial diagnostic instrument, enabling the choice of more targeted asthma treatments and improving overall disease management [10].

The study aims to evaluate whether introducing a single inhaler triple therapy with extra-fine high dose beclomethasone, formoterol, and glycopyrronium (BDP/FF/G) can improve the clinical, inflammatory, and functional condition of both large and small airways in patients with severe, uncontrolled asthma and SAD phenotype. Specifically, we aimed to examine whether simply switching from a conventional “open” triple therapy regimen to a “closed” single-inhaler extra-fine formulation on patients with uncontrolled asthma and SAD, who are candidates for biological therapy, can result in achieving quiet asthma by postponing treatment with biologicals.

## 2. Materials and Methods

### 2.1. Study Design

Our study was a prospective longitudinal investigation that included 32 patients from the Severe Asthma center at the Pulmonology Department of University Hospital Policlinico, Bari, Italy. Patient enrollment occurred from 1 March 2023 to 1 October 2023.

The study enrolled 32 participants aged 18 and above, diagnosed with confirmed severe uncontrolled asthma as per the Global Initiative for Asthma (GINA) step 5 guidelines [2]. For a minimum of a year, these individuals were undergoing a triple inhaled therapy regimen that included high-dose inhaled corticosteroids (ICS), delivered using at least two separate inhalation devices (known as “Open Inhalatory Triple Therapy”), without any past or present treatment with biological therapies. Eligibility required a SAD phenotype, indicated by either an IOS-measured fall in resistance from 5 to 20 Hz (R5–R20) exceeding 0.07 or spirometry %FEF25–75 below 65% [11].

Exclusion criteria were patients under 18, those unable to perform respiratory function tests, non-compliance with therapy, or having chronic obstructive pulmonary disease (COPD). Individuals with other lung conditions like cystic fibrosis (CF), CF-related bronchiectasis, allergic bronchopulmonary aspergillosis, positive tests for Aspergillus Fumigatus, pulmonary mycobacteriosis, previous pulmonary tuberculosis, or pulmonary fibrosis were also excluded.

**Figure 1:**
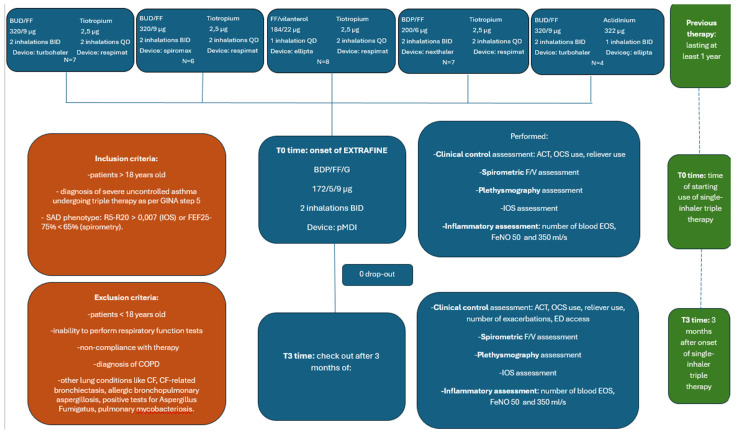
Flowchart describing the study design. Abbreviations: BUD, budesonide; FF, formoterol fumarate; BID, bis in die; QD, quam die; BDP, beclomethasone dipropionate; GINA, Global Initiative for Asthma; R5–R20, airway resistance from 5 to 20 Hz FEF25–75, forced expiratory flow between 25% and 75% of FVC; CF, cystic fibrosis; COPS, chronic obstructive pulmonary disease; p MDI, pressurized Metered Dose Inhaler; ACT, Asthma Control Test; EOS, eosinophilia; FeNO 50, fractional exhaled nitric oxide at 50 mL/s; FeNO 350, fractional exhaled nitric oxide at 350 mL/s.

Figure 1 outlines the study’s structure. We designated T0 Time as the start of the study, marking the transition from the “Open Inhalatory Triple Therapy” regimen, which participants had been on for a minimum of one year, to the initiation of a single-inhaler triple therapy regimen utilizing an extra-fine high dose of BDP/FF/G, referred to as “closed triple inhalation therapy”.

At T0, which serves as the baseline, patients had not yet commenced the single-inhaler triple therapy. T0 represents the initial point of assessment and spans a single day. During this day, all relevant parameters were collected from the patients. Following this assessment and collection of data, the single-inhaler triple therapy was prescribed to the patients.

Data collection occurred at the study’s outset, known as T0, and once more at 3 months (T3 time) after starting the single-inhaler triple therapy with extra-fine BDP/FF/G. This study was carried out following the principles of the Declaration of Helsinki, approved by a local Institutional Ethics Committee (Ethical Committee number: 6313, Approval date: 4 March 2020), and all subjects provided informed consent for study participation.

### 2.2. Patients

At the start of the study (T0), we collected demographic information, such as age and gender, along with smoking history and Body Mass Index (BMI). We phenotype asthma by noting its duration, age at diagnosis, and atopy presence. We also recorded the annual number of asthma exacerbations, emergency room visits in the past year, and the use of oral corticosteroids (OCS) and antibiotics. A broader patient profile included comorbidities like Eosinophilic Granulomatosis with Polyangiitis (EGPA), eosinophilic pneumonia, hyper-eosinophilic syndrome, nasal polyposis, urticaria, rhinosinusitis, vocal cord dysfunction, gastroesophageal reflux disease (GERD), obstructive sleep apnea syndrome (OSAS), and depressive-anxiety syndrome. At both T0 and after three months of therapy (T3), the following parameters were evaluated:

ACT and average daily reliever use over the last month, complete lung function evaluation, including Forced Expiratory Volume in one second (FEV1), Forced Vital Capacity (FVC), FEV1/FVC ratio, %FEV25–75, Total Resistance (R TOT), Total Lung Capacity (TLC), Residual Volume (RV), and RV/TLC ratio, Impulse Oscillometry (IOS) with calculation of: resistance at 5 Hz minus 20 Hz (R5–20), resonant frequency (Fres), reactance area (AX), reactance at 5 Hz (X5) and Inflammatory markers, including Fractional Exhaled Nitric Oxide at 50 mL/s (FENO 50) and at 350 mL/s (FENO 350).

### 2.3. Questionnaires

All patients in the study completed two questionnaires: the Asthma Control Test (ACT) [12] and the Test of Adherence to Inhalers (TAI) [13]. These tools were used to evaluate the level of asthma control and the extent of adherence to the prescribed inhaler treatment regimen.

### 2.4. Lung Function

Respiratory function tests for the enrolled patients were performed using the Masterlab Jaeger spirometer (Hoechberg, Germany), adhering to the European Respiratory Society/American Thoracic Society standards [14]. These tests included a forced expiratory maneuver to measure FEV1, FVC, the FEV1/FVC ratio (Tiffeneau index), and Forced Expiratory Flow at 25–75% of FVC (%FEF25–75) FeNO.

Additionally, patients underwent plethysmography to determine Total Lung Capacity (TLC), Residual Volume (RV), and respiratory system resistances, including Total Resistance (Rtot) and Effective Resistance (Reff) [15].

### 2.5. Impulse Oscillometry System (IOS)

IOS was performed using the MasterScreen IOS device manufactured by Hochberg, Germany, following the ERS protocol [16].

The technique involved having the patient breathe at tidal volume for approximately 30 s, while the operator held the subject’s cheeks with their hands to prevent vibrations from the device that affecting the maneuver.

The decrease in airway resistance from 5 to 20 Hz is used to assess resistance in the peripheral airways, with a threshold above 0.07 kPa × s × L^−1^ indicating SAD. Additional measurements include the reactance at 5 Hz (X5), indicating the elasticity of peripheral airways; the resonant frequency (Fres), where airway inertia and lung capacitance equalize; and the reactance area (AX), which reflects the elastic properties of the lung’s periphery and correlates with resistance at lower frequencies [17].

### 2.6. Measurement of Exhaled Nitric Oxide (FeNO) 50 and 350 mL/s

FeNO levels were measured at two different flow rates, 50 mL/s and 350 mL/s, utilizing an electrochemical-based analyzer, the FeNO+ by Medisoft-MGCD, Saint Paul, MN, USA. The measurement process was conducted in accordance with the manufacturer’s instructions and followed the guidelines recommended by the European Respiratory Society [18].

To accurately assess FeNO levels while eliminating the influence of nasal NO, a specialized breathing method was used. This method involved expiratory resistance and positive pressure within the mouth to maintain a steady expiratory flow at both 50 and 350 mL/s [19].

### 2.7. Statistical Analysis

We verified the distribution of the continuous variables under study using the one-sample Kolmogorov–Smirnov test. Continuous parametric variables were presented as mean (m) ± standard deviation (sd), whereas continuous non-parametric variables were shown as the median with the 25th and 75th interquartile ranges (IQ 25; IQ 75). The dichotomous or non-continuous variables were expressed as n (%). For continuous variables that are normally distributed, we conducted a Paired-Samples T Test to compare data from time T0 to T3 for the overall population. For data that did not follow a normal distribution, the Wilcoxon signed-rank test was utilized to compare pre- and post-values within groups. The McNemar test was used for the comparison of the discontinuous variables. All analyses were conducted with SPSS-26 (SPSS, Chicago, IL, USA). A significance value of *p* < 0.050 was assumed for all analyses.

## 3. Results

The average duration of the triple open inhalation therapy was 15.81 ± 3.20 months prior to transitioning to the closed inhalation therapy at time T0. The average duration of the triple open inhalation therapy was 15.81 ± 3.20 months prior to transitioning to the closed inhalation therapy at time T0.

The study population had an average age of 58.59 ± 15.03 years, with 53.1% females. The average Body Mass Index (BMI) was 26.65 ± 4.88. Among the participants, 59.4% were non-smokers and 40.6% were former smokers, with an average smoking history of 9.32 ± 16.02 pack-years. The average duration since asthma onset was 21.28 ± 14.23 years, and the average age at diagnosis was 26.75 ± 16.78 years. A significant majority (81.3%) of the patients were atopic (Table 1).

At the baseline measurement (T0), the yearly count of acute exacerbations was 2.78 ± 2.82; the number of oral corticosteroid (OCS) cycles was 1.68 ± 1.90, with an average dose per cycle of 17.65 ± 12.88. Emergency room visits averaged 0.25 ± 0.50 per patient.

In terms of baseline therapy in the year prior to the study, 37.5% of patients had undergone one cycle of antibiotics, and 9.4% had undergone two cycles. Anti-leukotrienes were prescribed to 12.5% of patients. The most common comorbidities were Gastroesophageal Reflux Disease (GERD) at 28.1%, nasal polyps at 21.9%, and Obstructive Sleep Apnea (OSA) at 18.8%. Additionally, 12.5% of patients had rhinosinusitis, 9.4% had Eosinophilic Granulomatosis with Polyangiitis (EGPA), bronchiectasis, anxiety-depressive syndrome, vocal cord dysfunction, and 6.3% had urticaria (see Table 1).

Table 2 highlights significant improvements in patient outcomes at T3. The ACT scores rose notably from an average of 15.75 ± 5.92 to 22.53 ± 1.45, indicating better asthma control (*p*-value < 0.001).

There were also significant enhancements in lung function, as shown by the increase in %FEV25–75 from 48.53 ± 13.31 at T0 to 63.62 ± 13.46 at T3 (*p* < 0.001), and a decrease in residual volume percentage (%RV) from 165.70 ± 63.51 at T0 to 141.65 ± 52.11 at T3 (*p* = 0.001).

Impulse Oscillometry (IOS) parameters showed marked improvement, reflecting reduced airway resistance. Notable changes include R5–20 decreasing from 0.14 (0.08; 0.29) at T0 to 0.07 (0.04; 0.09) at T3 (*p* < 0.001), Fres from 20.90 (17.69; 24.14) to 16.33 (12.77; 18.38) (*p* < 0.001), AX from 1.25 (0.48; 1.18) to 0.77 (0.43; 1.30) (*p* < 0.001), and X5 −1.20 (−1.47; −0.9) to −0.55 (−0.80; −0.50) (*p* < 0.001).

**Figure 2:**
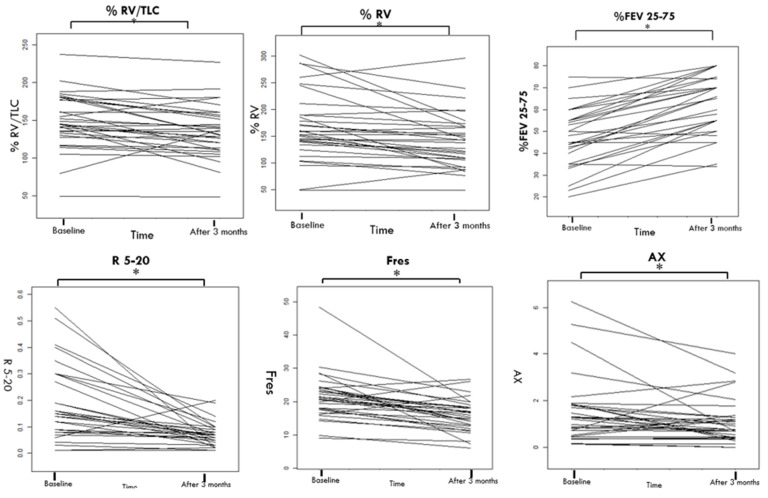
Small Airways Functional Control before and After 3 Months of High-Dose BDP/FF/G Triple Therapy. Abbreviations: FEF25–75, forced expiratory flow between 25% and 75% of FVC; TLC, total lung capacity; RV, Residual volume; R5–R20, airway resistance from 5 to 20 Hz; Fres, resonance frequency; AX, reactance area; X5, reactance at 5 Hz. *: *p* value < 0.050.

**Figure 3:**
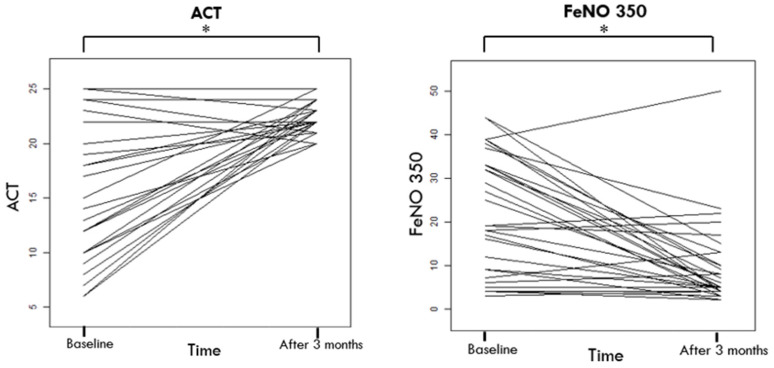
Clinical and Inflammatory improvement after 3 Months of High-Dose BDP/FF/G Triple Therapy. Abbreviations: ACT, Asthma Control Test; FeNO 350, fractional exhaled nitric oxide at 350 mL/s. *: *p* value < 0.050.

**Figure 4:**
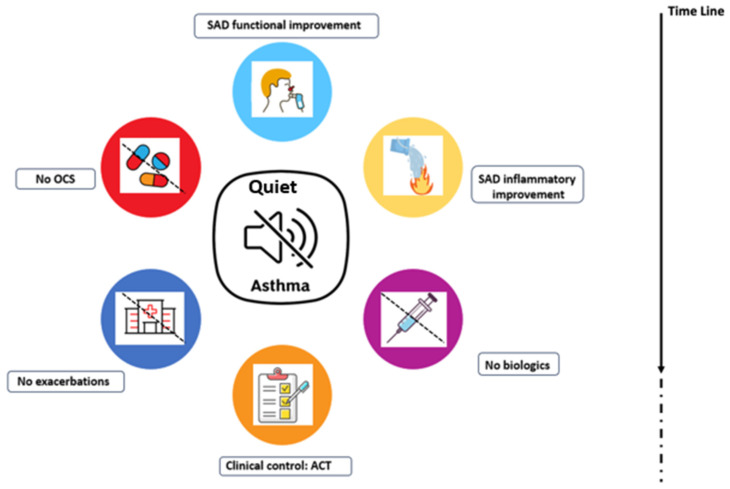
Quiet Asthma. Quiet Asthma is a comprehensive goal targeting patients with severe asthma and SAD phenotype prior to starting biological therapy. It aims for simultaneous outcomes: no need for oral corticosteroids, absence of exacerbations, improved ACT scores, reduced inflammation in SAD (evaluated using FeNO 350), and enhanced SAD functionality. Features of Quiet Asthma. Abbreviations: SAD, Small Airways Disease; OCS, oral corticosteroids; ACT, Asthma Control Test.

Additionally, there was a significant drop in FeNO at 350 mL/s, from 21.81 ± 13.83 at T0 to 9.31 ± 9.44 at T3, indicating reduced airway inflammation (see Table 2 and Figure 2, Figure 3 and Figure 4).

In a supplementary file, we conduct a comparative analysis between the baseline (T0) and the endpoint (T3) for each distinct therapeutic approach taken prior to T0 within the “Open Inhalatory Triple Therapy” category (Acl+ FF/BUD Turb: n = 4; Acl + FF/BUD Turb: n = 7; Tio + FF/BUD Spir: n = 6; Tio + Ume/Vil Ell: n = 8; Tio + FF/BDP Nex: n = 7). For details, refer to Supplementary Table S1, Figures S1 and S2.

## 4. Discussion

To our knowledge, our study is the first to demonstrate, in a group of 32 severe asthma patients with a SAD phenotype, who had suboptimal clinical control (ACT score below 20) and not yet undergoing biological therapy, that switching from a traditional “open” triple therapy regimen using two separate inhalers (ICS/LABA and LAMA) to a unified “closed” single-inhaler extra-fine formulation over a three-month period enhances small airway function, reduces small airway inflammation, and improves overall clinical control, leading to what we describe as a “Quiet Asthma” state. The proposed concept of “Quiet Asthma” represents a comprehensive pre-biological treatment objective for patients with severe, uncontrolled asthma displaying the SAD phenotype. This composite goal involves a consistent improvement in both the inflammation and functionality of the small airways, coupled with optimal symptoms control, no exacerbations, and the elimination of the need for oral corticosteroids (Figure 4).

Asthma, a common bronchial obstruction disorder, involves the entire bronchial system. The Small Airway constitutes over 98% of the lung’s surface area while accounting for less than 10% of its volume [20]. The small airways, or the “silent zone” (<2 mm diameter), significantly contribute to airway resistance in such conditions. Their inflammation or reaction to irritants leads to narrowing, while structural changes can alter their elasticity, affecting airflow dynamics [21]. Impairment in small airways is a common feature throughout all stages of asthma, affecting 50% to 60% of individuals with the condition [22].

In the ATLANTIS study involving 773 patients [9], the prevalence of SAD reached 91%, regardless of asthma control or severity, attributed to thorough SAD diagnostic methods. Over a year, SAD was detected using various indicators: abnormalities in FEF 25–75, lung volume deviations via body plethysmography (e.g., high RV/TLC), impulse oscillometry changes indicating resistance in smaller airways (R5–R20 difference), lung periphery distensibility (AX), and airway elasticity and inertia (X5). Additionally, some study assessed airway ventilation in conducting (Scond) and acinar (Sacin) airways through multiple breath nitrogen washout (MBNW). The Atlantis study highlights the importance of screening for SAD, particularly in patients with severe asthma, using all available diagnostic tools [9].

Given the high occurrence of SAD in asthma patients, which becomes more common as asthma severity increases [9], it is very likely to encounter the SAD phenotype in cases of severe asthma in clinical practice. In light of SAD’s prevalence, the study focused on severe, uncontrolled asthma patients with the SAD phenotype to assess whether enhancing small airway functionality could improve disease control and possibly delay the need for biological therapy. In our study of 32 participants, all were selected based on their SAD phenotype, characterized by R5–20 values above 0.07 and %FEV25–75 less than 65%. These participants were previously on high-dose ICS/LABA + LAMA through two separate inhalers, known as ‘open therapy’, yet they exhibited poor clinical control making them eligible for biological treatments according to GINA guidelines [2].

Switching to a single inhaler triple therapy with extra-fine beclometasone, formoterol, and glycopyrronium (BDP/FF/G) for three months significantly improved disease control in our study group. ACT scores rose notably, reflecting enhanced disease management. Concurrently, we have observed an improvement in the inflammation of the small airways assessed by FeNO 350 and in the function of the small airways with all the indicators under study (%FEV25–75, R5–20 Hz, X5, Fres, AX).

The addition of LAMA to a regimen of ICS/LABA has been extensively validated by research to improve pulmonary function, reduce the frequency of asthma exacerbations, and marginally enhance control in patients with moderate to severe asthma inadequately controlled by ICS-LABA alone [23,24]. LAMAs are effective across various asthma phenotypes and endotypes, with studies highlighting differences in onset and duration of action among tiotropium (TIO), glycopyrronium (GLY), and umeclidinium, where glycopyrronium starts working quicker than tiotropium [25].

A novel treatment approach involves a single inhaler delivering a triple therapy of extra-fine BDP/FF/G with particles <2 µm, improving small airway delivery [21]. This combination has shown a synergistic effect on bronchial relaxation in medium and small airways in ex vivo studies, attributed to the activation of glucocorticoid receptors and the Gsα subunit of β2-adrenoceptors, affecting the cyclic AMP-dependent PKA pathway. These findings need further clinical validation in asthma patients [26].

In our study group, we observed simultaneous improvements in Small Airways Dysfunction and ACT scores without significant changes in FEV1, which remained unchanged. Spirometry primarily indicates the variability and/or reversibility of airway obstruction but is not very effective at sensitively assessing small airways neither is predictive of clinical control [27]. It typically only shows abnormalities when around 75% of the small airways are obstructed [27]. A wealth of studies and systematic reviews have shown that SAD is linked to increased bronchial hyperreactivity, worse control of asthma, and more severe asthma flare-ups [9,10,28].

Various methods have been suggested for evaluating the small airways, with the initial approach being the measurement of %FEF25–75 through basic spirometry. Nonetheless, there is a lack of agreement on the dependability of %FEF25–75 in evaluating small airway function [29]. Recent advancements have led to the creation of more precise tests for accurately diagnosing SAD. Among these, IOS has emerged as a significant method for assessing lung function. IOS is an effort-independent technique that relies on the established Forced Oscillation Technique (FOT) [30].

In research by Cottini and colleagues [11], SAD was found to be independently associated with controlled asthma (Odds Ratio, 0.22; [0.06–0.84]), even in participants with normal spirometry Flow/Volume curves. This study, involving 321 asthma patients, examined the impact of SAD, as detected by IOS, on asthma management showing a strong association between the improvement of small airways function and clinical outcomes absolutely in line with our results [11].

Therefore, prior to biological therapy for patients with uncontrolled Severe Asthma exhibiting a SAD phenotype, achieving “Quiet Asthma” appears feasible. This composite outcome encompasses simultaneous improvement in both the inflammation and functionality of the small airways, clinical stability, absence of exacerbations, and the elimination of the requirement for oral corticosteroids. This allows us to move towards a precision medicine approach with the inclusion of inhaled therapies.

Furthermore, advancements in biologics for severe asthma are focused on achieving clinical remission, which several consensuses define as the absence of symptoms, stable lung function, and cessation of oral corticosteroids after one year [31,32,33]. The “Super Responder” (SR) concept, introduced by John W. Upham et al., identifies patients with significant biologic benefits using three criteria, including major improvements like no exacerbations and better asthma control. Superior responses appear early, while full remission may take longer [34]. A broader perspective on the SR concept underscores the importance of evaluating both biological and functional aspects, with a focus on the improvement of small airway function [35]. Thus, researching and treating the ‘silent zone’ in patients with severe asthma can potentially lead to a condition referred to as ‘Quiet Asthma’.

The primary limitations of our study include the small sample size and a brief follow-up period of just three months. Consequently, the duration of the “Quiet Asthma” state remains uncertain. For this reason, we are studying the same patients over a longer follow-up period of one year. Furthermore, this study, employing a pre-post within-group design, lacks the randomization and control group inherent to randomized controlled trials (RCTs), introducing potential selection and observational biases. The inclusion of subjects using two different devices (LAMA and LABA + ICS) without a control group further limits direct comparability and generalizability of the findings. These limitations are acknowledged, and the results should be interpreted within this context. Another study’s limitation centers on the numerous alterations introduced during the transition to single-inhaler triple therapy, including changes in inhalers, LAMA types, and ICS types, complicating the identification of the primary factor responsible for the enhanced outcomes. Despite this complexity, the results, especially the efficacy of a specific therapy regimen, are significant. Future investigations should aim to improve data clarity and explore the reasons for these therapeutic improvements more thoroughly, potentially by expanding the study sample size to strengthen the findings.

In conclusion, our study highlights the importance of investigating and treating less visible areas of the lung, such as Small Airway Dysfunction. By effectively “closing” the inhalation therapy and starting high-dose extra-fine BECL/GLIC/FORM inhalation treatment, we achieved a state of “Quiet Asthma”.

Future studies, involving larger cohorts and longer follow-up periods of at least a year, are essential to validate our findings and potentially ascertain the longevity of the Quiet Asthma condition.

## Figures and Tables

**Table 1:** Baseline characteristics of the enrolled population.

Demographic Parameters	
Age (yrs) m ± sd	58.59 ± 15.03
Sex Female n (%)	17 (53.1)
Life Style	
BMI (kg/m^2^) m ± sd	26.65 ± 4.88
Smoke n (%) No Ex	19 (59.4) 13 (40.6)
Pack/year m ± sd	9.32 ± 16.02
Characteristics of asthma	
Atopic yes n (%)	26 (81.3)
Duration of disease years m ± sd	21.28 ± 14.23
Age of diagnosis (years) m ± sd	26.75 ± 16.78
Exacerbations last year (n) m ± sd	2.78 ± 2.82
First Aid visits last year (n) m ± sd	0.25 ± 0.50
Average reliever usage last month (n) m ± sd	1.03 ± 1.59
OCS cycles last year (n) m ± sd	1.68 ± 1.90
OCS dose last year (mg) m ± sd	17.65 ± 12.88
Baseline Therapy	
Course of antibiotics last year n (%) 0 1 2	17 (53.1) 12 (37.5) 3 (9.4)
LTRA yes n (%)	4 (12.5)
Comorbidities	
EGPA yes n (%)	3 (9.4)
Eosinophilic Pneumonia yes n (%)	0 (0)
Hypereosinophilic syndrome yes n (%)	0 (0)
Rhinosinusitis yes n (%)	4 (12.5)
Nasal Polyposis yes n (%)	7 (21.9)
Urticaria yes n (%)	2 (6.3)
Vocal Cord Dysfunction yes n (%)	3 (9.4)
COPD yes n (%)	0 (0)
Bronchiectasis yes n (%)	3 (9.4)
GERD yes n (%)	9 (28.1)
OSAS yes n (%)	6 (18.8)
Depressive Anxious Syndrome yes n (%)	3 (9.4)

Abbreviations: BMI, Body Mass Index; OCS, oral corticosteroids; LTRA, Leukotriene Receptor Antagonist; EGPA, Eosinophilic Granulomatosis with Polyangiitis; GERD, gastro-esophageal reflux; COPD, Chronic Obstructive Pulmonary Disease; OSAS, Obstructive Sleep Apnea Syndrome; m ± sd, media ± standard deviation; n, number.

**Table 2:** Comparison of the asthmatic population (n = 32) in open triple inhaled therapy before and after the introduction of single device triple inhaled therapy.

Parameters	T0 Time	T3 Time	*p* Value
ACT m ± sd	15.75 ± 5.92	22.53 ± 1.45	<0.001
Average reliver usage last month (n) m ± sd	1.03 ± 1.59	0.25 ± 0.76	0.008
%FEV1 m ± sd	73.20 ± 22.86	73.90 ± 24.06	0.843
FEV1 (l) m ± sd	2.27 ± 0.99	2.30 ±0.99	0.736
%FVC m ± sd	84.85 ± 19.57	86.37 ± 15.65	0.623
FVC (l) m ± sd	3.21 ± 0.79	3.41 ± 1.14	0.199
%FEV1/FVC m ± sd	67.16 ± 12.75	67.88 ± 12.49	0.698
%FEV25–75 m ± sd	48.53 ± 13.31	63.62 ± 13.46	<0.001
%Rtot m ± sd	145.80 ± 86.72	151.35 ± 67.05	0.739
%TLC m ± sd	106.47 ± 22.38	101.78 ± 22.01	0.101
TLC (l) m ± sd	6.24 ± 1.16	5.84 ± 1.19	0.058
%RV m ± sd	165.70 ± 63.51	141.65 ± 52.11	0.001
RV (l) m ± sd	3.24 ± 1.29	2.86 ± 0.94	0.008
%RV/TLC m ± sd	147.58 ± 37.06	135.09 ± 34.38	0.002
RV/TLC m ± sd	51.61 ± 14.37	46.24 ± 14.94	0.003
R5–20 kPa·L^−1^·s^−1^ IQ (25; 75)	0.14 (0.08; 0.29)	0.07 (0.04; 0.09)	<0.001
Fres Hz IQ (25; 75)	20.90 (17.69; 24.14)	16.33 (12.77; 18.38)	<0.001
AX kPa/L IQ (25; 75)	1.25 (0.48; 1.18)	0.77 (0.43; 1.30)	0.009
X5 kPa·L^−1^·s^−1^ IQ (25; 75)	−1.20 (−1.47; −0.9)	−0.55 (−0.80; −0.50)	<0.001
Eosinophils (n/μL) m ± sd	262.59 ± 276.80	250.21 ± 259.29	0.160
FeNO 50 (ppb) m ± sd	12.28 ± 11.76	13.96 ± 16.84	0.639
FeNO350 (ppb) m ± sd	21.81 ± 13.83	9.31 ± 9.44	<0.001

Abbreviations: ACT, Asthma Control Test; FEV1, forced expiratory volume in the 1st second; FVC, forced vital capacity; FEF25–75, forced expiratory flow between 25% and 75% of FVC; TLC, total lung capacity; Rtot, total resistance; RV, Residual volume; R5–R20, airway resistance from 5 to 20 Hz; Fres, resonance frequency; AX, reactance area; X5, reactance at 5 Hz; Eos, eosinophilia; FeNO 50, fractional exhaled nitric oxide at 50 mL/s; FeNO 350, fractional exhaled nitric oxide at 350 mL/s; m ± sd, mean ± standard deviation; n, number; IQ 25–75, interquartile 25–75; kPa·L^−1^·s^−1^, kiloPascal per liter/second; kPa/L, kiloPascal per liter; Hz, Hertz; kPa·L^−1^·s^−1^, kiloPascal per liter/second; ppb, parts per billion. Data are displayed as n (%) or mean ± SD or median IQ 25–75.

## Data Availability

Data can be available at request.

## Supplementary Materials

The following supporting information can be downloaded at: https://www.mdpi.com/article/10.3390/jcm13082320/s1, Table S1. Subgroup Analysis: Assessing T0 versus T3 within Each Subgroup Utilizing a Specific Combination of Open Triple Therapy; Figure S1. Clinical and Inflammatory change from time T0 to T3 for each subgroup; Figure S2. Functional control of Small Airway Disease from time T0 to T3 for each subgroup.
