# Supplementary material for: Managing Small Airway Disease in Patients with Severe Asthma: Transitioning from the “Silent Zone” to Achieving “Quiet Asthma”
Source: J Clin Med. 2024 Apr 17;13(8):2320. doi: 10.3390/jcm13082320 (PMC11051485; doi:10.3390/jcm13082320)
Supplement: Supplementary file 1 [file jcm-13-02320-s001.zip › jcm-2920732-supplementary.pdf]

Supplemental Table S1. Subgroup Analysis: Assessing T0 versus T3 within Each Subgroup Utilizing a Specific Combination of Open Triple Therapy.

	**Bud /form Turb+ Acl N= 4**			**Bud /form Turb + Tio N= 7**			**Bud /form Spir + Tio N=6**			**Ume/Vil Ell + Tio N=8**			**BDP/FF Nex + Tio N=7**		
**Parameters**	**TO**	**T3**	**P value**	**TO**	**T3**	**P value**	**TO**	**T3**	**P value**	**TO**	**T3**	**P value**	**TO**	**T3**	**P value**
ACT m ± sd	19±4.54	22.25±2.21	0.090	13.85±5.01	23.14±1.06	0.004	15.16±6.61	22.66±1.21	0.052	17.25±6.43	22.62±1.92	0.034	14.57±6.72	21.85±0.89	0.039
Average reliver usage last month (n) m ± sd	0.25±0.5	0.5±0.57	0.391	2.85±1.77	0.57±1.51	0.022	0.16±0.40	0.00±0.00	0.363	0.62±1.40	0.00±0.00	0.250	0.85±1.46	0.28±0.48	0.356
%FEV1 m ± sd	85.42±26.25	74.25±28.21	0.380	70.3±23.05	68.42±13.90	0.743	79.5±30.76	77.33±33.80	0.553	63.21±15.17	75.01±23.29	0.290	21.90±8.27	27.68±10.46	0.975
%FVC m ± sd	101.05 ±30.84	95±10.03	0.710	74.11±16.22	75.42±12.86	0.815	89.83±21.88	84.83±15.27	0.391	78.8±14.78	88.92±10.53	0.205	89.01±13.54	90.8±22.41	0.638
%FEV1/FVC m ± sd	65.19±6.33	66.32±4.33	0.717	69.21±16.55	68.06±12.70	0.801	69.99±16.45	69.48±12.82	0.821	64.42±12.92	68.71±17.64	0.444	66.92±9.99	66.27±11.28	0.869

%FEV 25-75 m ± sd	43.50±13.12	68.75±13.14	0.005	50.71±65.57	13.97±16.24	0.007	47.16±13.22	61.33±11.34	0.003	50±16.32	63.87±15.68	0.004	48.71±12.25	60.42±12.36	0.010
%Rtot m ± sd	111±33.49	180.25±120.66	0.252	172.85±159.21	141.57±44.60	0.619	116.25±58.35	141±55,23	0.175	125.46±36.08	118.40±54.00	0.736	187.22±58.47	191.14±62.81	0.092
%TLC m ± sd	123.72±16.75	108.32±24.39	0.314	96.92±11.25	89.42±14.61	0.203	101.5±35	93±26.74	0.146	113.47±23.60	119.15±20.73	0.323	102.41±15.38	98.10±13.88	0.334
%RV m ± sd	172.32±55.68	136.25±26.13	0.196	153.04±51.98	132.14±50.29	0.190	159.85±111.47	121.33±50.08	0.214	196.5±53.48	182±63.82	0.151	151.27±25.97	125.54±36.14	0.027
%RV/TLC m ± sd	136.62 ±26.13	132±34.72	0.309	152.05±44.82	138.27±43.38	0.091	136.75±62.27	124±40.40	0.486	162.62±23.24	146.41±24.90	0.009	147.74±7.29	130.24±34.66	0.166
R5-20 kPa·L^{-1}·s^{-1} IQ (25 ; 75)	0.28 (0.11 ; 0.30)	0.09 (0.03 ; 0.18)	0.144	0.12 (0.09;0.12)	0.06 (0.04;0.07)	0.018	0.11(0.05;0.24)	0.06(0.01;0.09)	0.042	0.11(0.04;0.31)	0.06(0.02;0.08)	0.028	0.16 (0.15;0.40)	0.1(0.07;0.14)	0.018
Fres Hz IQ (25 ; 75)	23.10 (19.10 ; 24.01)	17.57 (14.79 ; 24.62)	0.273	18.03 (15.82; 24.58)	13(11.4;14.05)	0.018	22.84(17.74;27.29)	16.89(13.56;2075)	0.027	20.78(16.02;27.34)	15.31(8.11;19.5)	0.093	21(16.68;22.88)	18.1(15.0;19.0)	0.176
AX kPa/L IQ (25 ; 75)	1.98 (0.72 ; 3.91)	0.80 (0.45 ; 2.35)	0.465	0.99 (0.83;1.86)	0.7(0.35;1.01)	0.128	1.03(0.36;2.29)	0.62(0.38;0.62)	0.116	1.01(0.22;1.44)	0.76(0.11;2.39)	0.161	1.7(0.51;1.9)	1.3(1.2;1.78)	0.612

0.038X5 kPa·L^{-1}·s^{-}IQ (25 ; 75)	-1.21 (-1.65 ; -1.20)		0.068	-1.23 (-1.4;-0.9)	-0.5(-0.8;-0.5)	0.018	-1.15(-2.1;-0.72)	-0.65(-0.92;-0.32)	0.028	-1.05(-1.72;-0.75)	-0.55(-0.87;-0.50)	0.011	-1.1(-1.3;-1.01)	-0.8(-0.0;-0.5)	
Eosinophils (n/µl) m ± sd	326.66±111.50	350.00±145.25	0.689	240±235.23	210±206.63	0.247	187±40.40	193.33±85.18	0.470	268±352.31	248.5±316.74	0.213	351±397.28	328.42±382.53	0.339
FeNO 50 (ppb) m ± sd	11.75±4.57	30.50±41.29	0.429	10.85±7.47	10.86±7.76	0.99	15.66±19.89	8.5±3.37	0.342	13.75±15.50	10.25±611	0.592	9.42±3.45	16.57±15.76	0.269
FeNO350 (ppb) m ± sd	24.25±14.77	20±20.63	0.647	22.57±7.85	15.39±4.81	0.014	19.5±15.78	6.83±6.67	0.121	20-75±14.29	7.75±7.09	0.039	22.85±13.59	8.57±6.34	0.035

Abbreviations: BUD, budesonide; FF, formoterol fumarate; Turb, turbohaler; Tio, tiotropium Respimat; Acl, aclidinium Genuair; Spir, spiromax; BID, bis in die; QD, quam die; BDP, beclomethasone dipropionate; Ume/Vil Ell, Umeclidinium/Vilanterolo Ellipta; Nex, Nexthaler; GINA, Global Initiative for Asthma ACT, Asthma Control Test; FEV1, forced expiratory volume in the 1st second; FVC, forced vital capacity; FEF25–75, forced expiratory flow between 25% and 75% of FVC; TLC, total lung capacity; Rtot, total resistance; RV, Residual volume; R5-R20, airway resistance from 5 to 20 Hz; Fres, resonance frequency; AX, reactance area; X5, reactance at 5 Hz; Eos, eosinophilia; FeNO 50, fractional exhaled nitric oxide at 50 ml/s; FeNO 350, fractional exhaled nitric oxide at 350 ml/s; m ± sd, mean ± standard deviation; n, number; IQ 25 75, interquartile 25 75; kPa·L^{-1}·s^{-1}, kiloPascal per litro al secondo; kPa/L, kiloPascal per litro; Hz, Hertz ; kPa·L^{-1}·s^{-1}, kiloPascal per litro al secondo; ppb, parts per billion . Data are displayed as n(%) or mean ± SD **or median IQ 25;75**

Suppl Figure S1. Clinical and Inflammatory change from time T0 to T3 for each subgroup

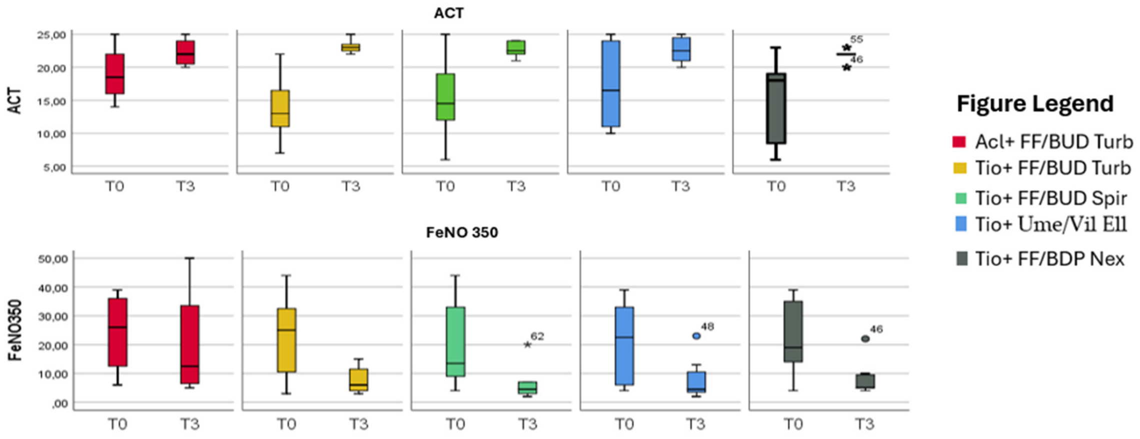

Acl+ FF/BUD Turb: n= 4; Acl+ FF/BUD Turb: n=7 ; Tio+ FF/BUD Spir: n=6 ; Tio+ Ume/Vil Ell: n=8; Tio+ FF/BDP Nex: n=7
Analysis within each subgroup defined based on the specific combination of open therapy. Clinical and Inflammatory change from time T0 to T3

Abbreviations: BUD, budesonide; FF, formoterol fumarate; Turb, turbohaler; Tio, tiotropium Respimat; Acl, aclidinium Genuair; Spir, spiromax; BID, bis in die; QD, quam die; BDP, beclomethasone dipropionate; Ume/Vil Ell, Umeclidinium/Vilanterolo Ellipta; Nex, Nexthaler; GINA, Global Initiative for Asthma ACT, Asthma Control Test;; FeNO 350, fractional exhaled nitric oxide at 350 ml/s; Supl, supplementary

Figure S2. Functional control of Small Airway Disease from time T0 to T3 for each subgroup

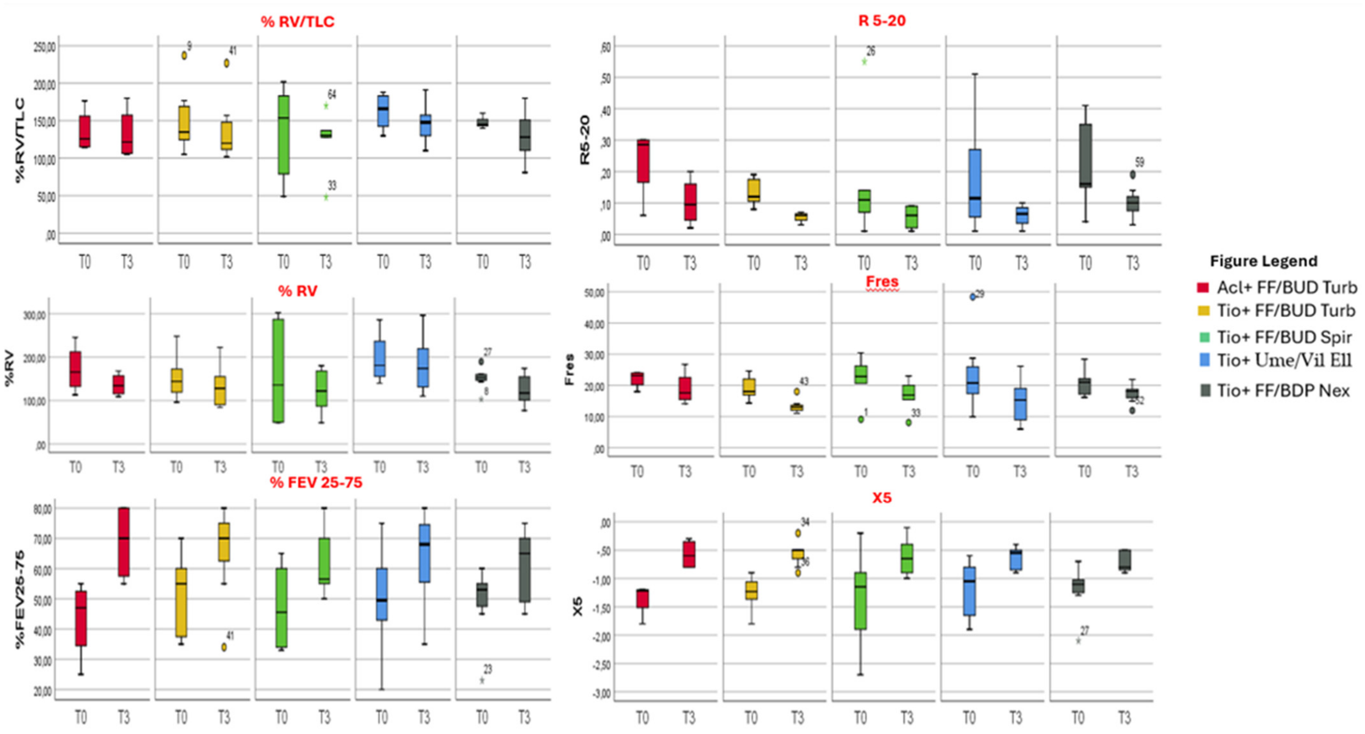

Acl+ FF/BUD Turb: n= 4; Acl+ FF/BUD Turb: n=7 ; Tio+ FF/BUD Spir: n=6 ; Tio+ Ume/Vil Ell: n=8; Tio+ FF/BDP Nex: n=7
Analysis within each subgroup defined based on the specific combination of open therapy.
Functional control of Small Airway Disease from time T0 to T3

Abbreviations: BUD, budesonide; FF, formoterol fumarate; Turb, turbohaler; Tio, tiotropium Respimat; Acl, aclidinium Genuair; Spir, spiromax; BID, bis in die; QD, quam die; BDP, beclomethasone dipropionate; Ume/Vil Ell, Umeclidinium/Vilanterolo Ellipta; Nex, Nexthaler; GINA, Global Initiative for Asthma ACT, Asthma Control Test; FEV1, forced expiratory volume in the 1st second; FVC, forced vital capacity; FEF25–75, forced expiratory flow between 25% and 75% of FVC; TLC, total lung capacity; Rtot, total resistance; RV, Residual volume; R5-R20, airway resistance from 5 to 20 Hz; Fres, resonance frequency; AX, reactance area; X5, reactance at 5 Hz; Eos, eosinophilia; FeNO 50, fractional exhaled nitric oxide at 50 ml/s; FeNO 350, fractional exhaled nitric oxide at 350 ml/s; m ± sd, mean ± standard deviation; n, number; IQ 25 75, interquartile 25 75; $kPa \cdot L^{-1} \cdot s^{-1}$, kiloPascal per litro al secondo; kPa/L, kiloPascal per litro; Hz, Hertz ; $kPa \cdot L^{-1} \cdot s^{-1}$, kiloPascal per litro al secondo; ppb, parts per billion . Data are displayed as n(%) or mean ± SD or median IQ 25;75; Supl, supplementary
